# Drug Interactions Lead to Recurrent Seizures in Brucellosis: A Case Report

**DOI:** 10.7759/cureus.83032

**Published:** 2025-04-26

**Authors:** Zhenglin Xiao, Qingzi Yan, Renzhu Liu

**Affiliations:** 1 Department of Pharmacy, Xiangtan Central Hospital (The Affiliated Hospital of Hunan University), Xiangtan, CHN; 2 School of Biomedical Sciences, Hunan University, Changsha, CHN; 3 Department of Clinical Pharmacy, Xiangtan Central Hospital (The Affiliated Hospital of Hunan University), Xiangtan, CHN

**Keywords:** adverse drug reaction, brucellosis, drug interaction, epilepsy, therapeutic drug monitoring

## Abstract

Brucellosis is a prevalent zoonotic infectious disease that poses a significant public health concern. The economic burden of human brucellosis includes hospitalization costs, pharmacological treatment, out-of-pocket expenses, and income loss due to missed work during illness. This case report describes recurrent seizures resulting from drug interactions in a patient undergoing treatment for brucellosis. Although rifampicin-based regimens are the first-line treatment for brucellosis, rifampicin is a potent inducer of various drug-metabolizing enzymes and can interact with numerous medications, including antiepileptic drugs. Alternative anti-Brucella agents may be considered when drug interactions are of concern. The choice of antiepileptic therapy should aim to minimize potential interactions with rifampicin. If such interactions are unavoidable, co-administration should be closely managed using therapeutic drug monitoring to prevent subtherapeutic dosing, seizure recurrence, or compromised brucellosis treatment efficacy. In this case, rifampicin was replaced with an alternative anti-Brucella agent to minimize interaction risks, and antiepileptic drug dosages were optimized using therapeutic drug monitoring to achieve seizure control. Patient education on medication adherence was also reinforced to support treatment outcomes.

## Introduction

Currently, human brucellosis remains a significant public health concern in China. Brucellosis is a common zoonotic infectious disease affecting approximately 500,000 people annually [1]. According to the Chinese Center for Disease Control and Prevention, 69,767 cases were reported in Mainland China in 2021, representing a 47.7% increase from the 47,425 cases reported in 2020 [2]. The most common mode of infection is the consumption of unpasteurized milk or dairy products. The typical clinical manifestation involves fever, accompanied by systemic symptoms. Neurological involvement occurs in 4−7% of cases, manifesting in diverse forms, including meningoencephalitis and peripheral nervous system involvement [3]. Bacterial culture of various body fluids is the gold standard for brucellosis diagnosis; however, the bacteria are obligately aerobic and exhibit slow growth, and significant growth may not be evident until after 72 hours of culture [4]. Prolonging hospital stays can enhance the positive culture rate. Consequently, clinical practice often utilizes the tube agglutination test (TAT) with a titer of ≥ 1:100 [5]. Treatment for human brucellosis includes doxycycline, rifampicin, aminoglycosides (streptomycin or gentamicin, rarely amikacin), trimethoprim/sulfamethoxazole (TMP-SMX), and quinolones (ciprofloxacin or ofloxacin) [6].

## Case presentation

A 22-year-old male patient was admitted to the hospital on June 21, 2024, with a history of recurrent fever lasting over five months. Upon admission, his temperature was 39.6°C, accompanied by chills, poor appetite, dry mouth, occasional dizziness and headaches, left hip pain, and difficulty walking. The patient had a history of brucellosis and epilepsy, likely due to exposure from raising sheep at home. He was prescribed carbamazepine, sodium valproate, and lamotrigine for epilepsy management, in addition to doxycycline and rifampin for brucellosis. However, his medication adherence was suboptimal, resulting in irregular drug intake.

Upon admission, laboratory investigations revealed elevated C-reactive protein (CRP) levels at 78.33 mg/L. The changes in laboratory parameters during the treatment of the patient are shown in Table 1.

**Table 1:** The laboratory examination during the diagnosis and treatment of the patient. BT, body temperature; WBC, white blood cell count; PLT, platelet count; NEU, absolute neutrophil count; NE%, neutrophil percentage; CREA, creatinine; CRP, C-reactive protein; VPA, valproic acid; LTG, lamotrigine; CBZ, carbamazepine.

Investigations	Day 1	Day 6	Day 10	Day 45	Reference range
BT (℃)	39.1	37.3	36.6	36.3	36.1-37.0
WBC (*10^9^/L)	3.90	7.90	5.40	4.67	3.97-9.15
PLT (*10^9^/L)	356.0	447.0	331.0	247.0	85.0-303.0
NEU (*10^9^/L)	2.33	5.83	2.90	2.15	2.0-7.0
NE%	59.7	73.9	53.6	46.0	50.0-70.0
CREA (μmol/L)	61.0	47.0	55.0	79.0	53.0-115.0
CRP (mg/L)	78.33	41.63	12.90	NA	0.00-3.00
VPA (mg/L)	NA	19.60	37.91	58.41	50.00-100.00
LTG (mg/L)	NA	0.51	1.09	9.42	2.50-15.00
CBZ (mg/L)	NA	1.59	5.75	NA	4.00-12.00

Initial treatment for brucellosis consisted of doxycycline 0.1 g orally twice daily (BID), rifampicin 0.6 g orally once daily (QD), and ceftriaxone 2 g intravenously every 12 hours (Q12H). For epilepsy management, he received sodium valproate 0.4 g BID, lamotrigine 50 mg BID, and carbamazepine 0.2 g BID. Given the persistent fever attributed to uncontrolled brucellosis, the rifampicin dosage was increased to 0.9 g QD the following day. On the fourth day of admission, the patient experienced two seizures. The first seizure presented as a slight twitching of both hands, without any upward eye deviation or foaming at the mouth, and lasted approximately five minutes. The second seizure occurred six hours later and was characterized by generalized convulsions accompanied by a loss of consciousness. In response to these events, diazepam was administered intramuscularly for sedation, along with oxygen inhalation. Following this intervention, the patient's epileptic symptoms were alleviated. Notably, the interval between seizures was reduced compared to the previous baseline level. Considering the potential drug interaction between rifampicin and the antiepileptic medications, rifampicin was discontinued and replaced with oral levofloxacin tablets. Plasma concentrations of the antiepileptic drugs were monitored. Despite these interventions, the patient continued to experience seizures on the fifth day of admission.

**Figure 1:**
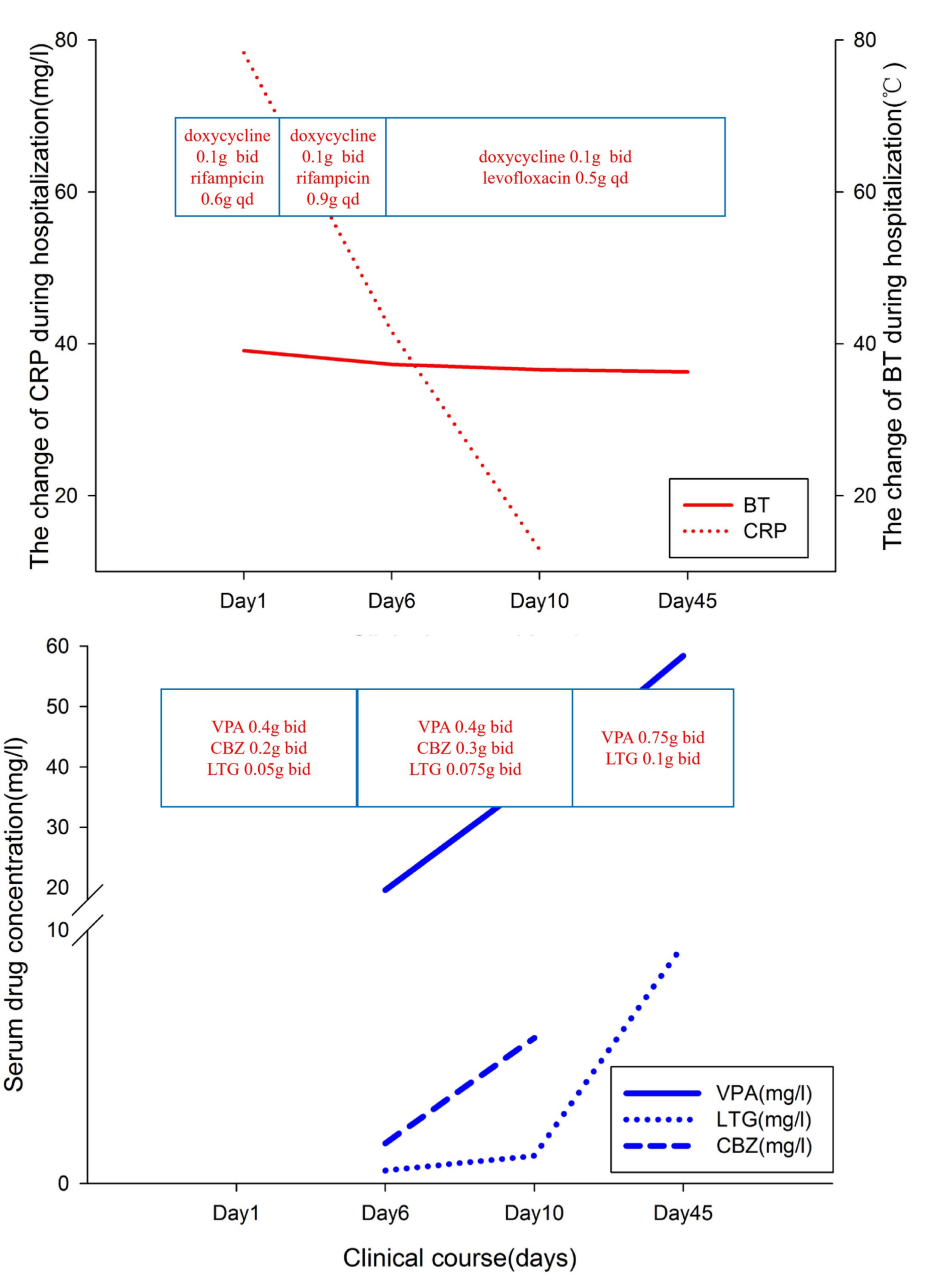
Changes in serum concentrations of antiepileptic drugs and infection indicators during the diagnosis and treatment of the patient. BT, body temperature; CRP, C-reactive protein; VPA, valproic acid; LTG, lamotrigine; CBZ, carbamazepine.

On the sixth day of admission, the plasma concentrations of antiepileptic drugs were as follows: lamotrigine: 0.51 mg/L; sodium valproate: 19.60 mg/L; and carbamazepine: 1.59 mg/L. The changes in serum concentrations of antiepileptic drugs during the patient's treatment are shown in Figure 1.

The dosages of carbamazepine and lamotrigine were subsequently adjusted to 0.3 g BID and 75 mg BID, respectively. On the seventh day of admission, the patient's body temperature had decreased to 37.3°C, and the CRP level was 41.63 mg/L, indicating a positive response to anti-infection and antiepileptic treatments. The current treatment regimen was continued. On the eighth day of admission, the frequency of seizures had decreased compared to the pre-adjustment period, and the concentrations of antiepileptic drugs were reassessed. On the 10th day of admission, the plasma drug concentrations were as follows: lamotrigine: 1.09 mg/L; carbamazepine: 5.75 mg/L; valproic acid: 37.91 mg/L; and CRP: 12.90 mg/L. By the 15th day of admission, the patient had no seizures and reported no significant discomfort. The discharge recommendations emphasized the importance of strict adherence to antiepileptic and anti-Brucella medications, following the prescribed dosage and schedule, and attending regular follow-up appointments. Following discharge, the patient continued anti-Brucella treatment at a community hospital for two weeks. Twelve days post discharge, after a period of prolonged work, the patient experienced episodic rigidity and transient impaired consciousness, which resolved spontaneously within minutes. The treatment regimen was modified to include valproate at a dosage of 0.75 g administered BID, in conjunction with lamotrigine at a dosage of 100 mg BID. The antiepileptic drug regimen was adjusted to incorporate valproic acid at 0.75 g BID, combined with lamotrigine at 100 mg BID. The dosages of both valproic acid and lamotrigine were increased to mitigate potential interactions between the antiepileptic medications, while carbamazepine was discontinued, followed by a review of antiepileptic drug plasma concentrations. One month post discharge, valproic acid levels were 58.41 mg/L and lamotrigine levels were 9.42 mg/L. The patient remained seizure-free over the subsequent one to two years, allowing for a gradual tapering of antiepileptic medications.

## Discussion

Human brucellosis is a significant zoonotic disease caused by bacteria of the genus Brucella. In 2021, the incidence rate of human brucellosis in China was 4.95 per 100,000 population. Brucellosis is primarily transmitted from infected livestock (sheep, goats, cattle, camels, and pigs) and wild animals through the consumption of raw dairy products, infected meat, and close contact with their secretions and carcasses. Therefore, the meticulous collection of medical history, evaluation of clinical manifestations, performance of auxiliary examinations, and consideration of epidemiological factors are crucial to differentiate it from other infectious diseases [7]. Doxycycline and rifampicin are commonly used antibiotics for the management of brucellosis and serve as the foundation for treating all forms of human brucellosis. The treatment principles include early intervention, combination therapy, adequate dosage, and completion of a full course of medication, with the potential extension of treatment duration to prevent recurrence and chronicity. Close monitoring of blood routines and liver and kidney function during treatment is essential. Preferred regimens for uncomplicated infections without complications in adults and children over eight years old include doxycycline (six weeks) combined with gentamicin (one week ), doxycycline (six weeks) with streptomycin (two to three weeks), or doxycycline (six weeks) with rifampicin (six weeks) [8]. In this case, the initial treatment regimen consisted of rifampin and doxycycline. However, due to suboptimal patient adherence and potential drug interactions, brucellosis was not adequately controlled, leading to recurrent episodes of fever.

Rifampicin is a potent inducer of the cytochrome P450 (CYP450) enzyme system, which can significantly enhance the expression and activity of various CYP450 enzymes, particularly CYP3A4. This induction may lead to an accelerated metabolism of drugs administered concurrently with rifampicin, potentially resulting in reduced blood concentrations and diminished efficacy of these medications. In the CYP450 enzyme system, enzymes such as CYP1A2, CYP2C9, CYP2C19, and CYP2D6 play crucial roles in drug metabolism. Additionally, rifampicin induces certain drug transporters, such as P-glycoprotein [9]. The induction of these enzymes and transporters by rifampicin could precipitate drug interactions, increase the risk of adverse reactions, or compromise therapeutic effectiveness.

Valproic acid is primarily metabolized by CYP450 enzymes. Specifically, CYP2C19 is involved in the metabolism of valproic acid, and its gene polymorphism can influence the blood concentration of valproic acid. Rifampicin may reduce the blood concentration of sodium valproate, potentially leading to reduced efficacy and consequently inducing seizures [10]. Therefore, when these two drugs are used concomitantly, timely monitoring of blood concentrations is necessary, and the dosage of sodium valproate should be adjusted if required to ensure the therapeutic effect. Rifampicin is an effective inducer of several drug-metabolizing enzymes belonging to the CYP450 superfamily and the uridine 5'-diphosphate (UDP)-glucuronosyltransferase system, and lamotrigine is mainly eliminated by metabolic inactivation by the latter. Drugs that affect UDP-glucuronosyltransferase activity can interact with lamotrigine [11]. In this case, the initial treatment regimen included rifampin combined with doxycycline for brucellosis and valproic acid and carbamazepine combined with lamotrigine for epilepsy. However, due to poor patient compliance and drug interactions, brucellosis was not completely controlled, as evidenced by repeated fever. Following admission, the antiepileptic serum concentrations were below the normal range. Considering the interaction between rifampicin and antiepileptic drugs, rifampicin was replaced with levofloxacin, and the dosage of antiepileptic drugs was appropriately increased. Over the subsequent days, the serum concentration of antiepileptic medications gradually increased, the frequency of seizures significantly decreased, and no fever occurred. The infectious markers showed a downward trend, indicating that the brucellosis infection was gradually being controlled.

## Conclusions

Rifampicin is the preferred treatment for brucellosis. However, in patients with secondary epilepsy or epilepsy resulting from central nervous system involvement, it is essential to avoid antiepileptic drugs that may interact with rifampicin. If avoidance is not feasible, dosing regimens for both rifampicin and the antiepileptic medication should be adjusted under the guidance of therapeutic drug monitoring. This approach aims to prevent seizures due to inadequate dosing or failure to treat brucellosis effectively.
